# Bioactive Bismuth Compounds: Is Their Toxicity a Barrier to Therapeutic Use?

**DOI:** 10.3390/ijms25031600

**Published:** 2024-01-27

**Authors:** Ângela Gonçalves, Mariana Matias, Jorge A. R. Salvador, Samuel Silvestre

**Affiliations:** 1 CICS-UBI—Health Sciences Research Centre, University of Beira Interior, Av. Infante D. Henrique, 6201-506 Covilhã, Portugal; angela.goncalves@ubi.pt (Â.G.); mariana.matias@fcsaude.ubi.pt (M.M.); 2 Laboratory of Pharmaceutical Chemistry, Faculty of Pharmacy, University of Coimbra, 3000-548 Coimbra, Portugal; salvador@ff.uc.pt; 3 CNC—Centre for Neuroscience and Cell Biology, University of Coimbra, 3004-517 Coimbra, Portugal

**Keywords:** bismuth compounds, toxicity, therapeutic properties, bismuth iodoform paraffin paste, bismuth overuse

## Abstract

Bismuth compounds are considered relatively non-toxic, with their low solubility in aqueous solutions (e.g., biological fluids) being the major contributing factor to this property. Bismuth derivatives are widely used for the treatment of peptic ulcers, functional dyspepsia, and chronic gastritis. Moreover, the properties of bismuth compounds have also been extensively explored in two main fields of action: antimicrobial and anticancer. Despite the clinical interest of bismuth-based drugs, several side effects have also been reported. In fact, excessive acute ingestion of bismuth, or abuse for an extended period of time, can lead to toxicity. However, evidence has demonstrated that the discontinuation of these compounds usually reverses their toxic effects. Notwithstanding, the continuously growing use of bismuth products suggests that it is indeed part of our environment and our daily lives, which urges a more in-depth review and investigation into its possible undesired activities. Therefore, this review aims to update the pharmaco-toxicological properties of bismuth compounds. A special focus will be given to in vitro, in vivo, and clinical studies exploring their toxicity.

## 1. Introduction

The term bismuth originates from the German word Weissmuth or Wismut, meaning white substance. Bismuth is the heaviest stable element, has an atomic mass of 208.980, is the 83rd element of the periodic table, and is also the least abundant of the elements of the Group 15 [1,2]. Often classified as a semi-metal or metalloid, bismuth has the characteristics of a metal and has properties identical to semiconductors and insulators. As somewhat of a rare element, bismuth’s abundance is analogous to that of silver and mercury, even though it is cheaper because large amounts are recovered as a by-product of copper and tin refining [3].

Bismuth and bismuth compounds are thought of as moderately safe and fairly non-toxic despite being in close proximity in the periodic table with elements such as arsenic, antimony, lead, and tin, which are highly toxic and are associated with relevant environmental hazards. The relatively low toxicity of bismuth compounds has been mainly attributed to their insolubility in nearly neutral aqueous solutions, for example, biological fluids. Furthermore, it was demonstrated that most bismuth compounds have a lower toxicity than that of sodium chloride [3]. Nonetheless, the nature of the counter ion or ligands bound to bismuth clearly contributes to the toxicity of some bismuth compounds (e.g., BiPh_3_ and Bi(OTf)_3_·xH_2_O).

This relative safety is one of the reasons explaining why several bismuth-based chemical processes have been established and applied in the synthesis of biologically active natural products, active pharmaceutical ingredients, and important derivatives, and other medicinally and pharmaceutically relevant molecular structures. In fact, different bismuth derivatives are used in synthetic medicinal chemistry, namely as catalysts [4,5,6,7,8,9]. In addition, bismuth salts, particularly colloidal bismuth subcitrate and bismuth subsalicylate, are commonly used to treat peptic ulcers, functional dyspepsia, and chronic gastritis [10,11,12,13]. Moreover, the potential use of bismuth radionuclides (e.g., ^213^Bi) in the treatment of different tumors is of high importance, having reached clinical trials and being used in successful therapeutic strategies. Furthermore, various organobismuth compounds have also been studied over the years as possible antiulcer, antimicrobial, and anticancer agents. Interestingly, bismuth is also used in the cosmetic industry as a component of some pigments [14].

These features of bismuth compounds justify their high interest in the fields of organic chemistry, pharmacy, and medicine [15,16,17,18,19,20,21]. Therefore, it is important to review the available toxicological data regarding this element to contribute to a better assessment of its safety in the numerous applications of bismuth-based products. In fact, notwithstanding the many beneficial qualities of bismuth, various side effects, including neurological syndromes, have been reported. However, bismuth toxicity may mainly be developed due to ingestion in extreme doses, or incorrect use when taken in large quantities and for an extended period of time [22]. The reported toxic effects that were reported as caused by an overdose of bismuth compounds comprise encephalopathy, nephropathy, osteoarthropathy, gingivostomatitis, and colitis [23]. Indeed, bismuth poisoning typically disturbs the kidney, liver, and bladder, among other organs. Interestingly, nephrotoxicity is mainly attributed to acute toxicity, while chronic exposure to high levels of bismuth compounds can often result in encephalopathy [24]. In this context, the best documented case of bismuth neurotoxicity was the occurrence of bismuth encephalopathy in several patients in France [25]. It is important to mention that signs of bismuth accumulation were observed in different cell types, including kidney cells [26], motor neurons [27], ganglion cells [28], and Leydig cells [29]. In all these cases, bismuth was found in the lysosomes, which contribute to heavy-metal metabolism. However, a decreased number of intact lysosomes were found, since intralysosomal bismuth induces lysosomal rupture [30]. 

In this review, the therapeutic properties and toxicity of different bismuth compounds will be updated, with a special focus on toxicological data from in vitro and in vivo studies. In addition, the adverse events reported for bismuth derivatives at the clinical set will also be included and discussed.

## 2. Bismuth Compounds with Therapeutic Properties

**Figure 1:**
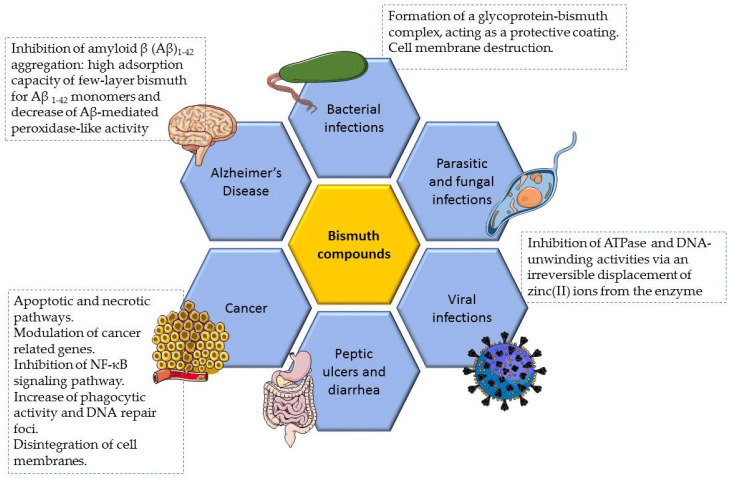
The general main therapeutic properties of bismuth compounds and the main putative mechanisms of action of different bismuth compounds. A portion of the scheme was developed by using pictures from Servier Medical Art. Servier Medical Art by Servier is licensed under a Creative Commons Attribution 3.0 Unported License: https://creativecommons.org/licenses/by/3.0/ (accessed on 22 September 2023).

Many bismuth compounds have been prepared and some of them have reached the market, being principally applied in the clinical field. Presently, bismuth is being therapeutically explored in two main fields: antimicrobial and anticancer. In fact, bismuth can interact with nucleotides and amino acids in enzymes and other proteins, which is closely related to its uptake, accumulation, transport, and excretion in the human body, as well as to their antimicrobial and anticancer activities [31,32,33]. A summary of the biological properties of bismuth compounds as well as its main mechanisms of action are illustrated in Figure 1 and in Table S1 (Supplementary Materials).

### 2.1. Antiulcer and Anti-Infective Effects

Bismuth compounds have been utilized in the treatment of various gastrointestinal disorders and microbial infections, such as syphilis, colitis, wound infection, dyspepsia, diarrhea and peptic ulcers [34].

**Figure 2:**
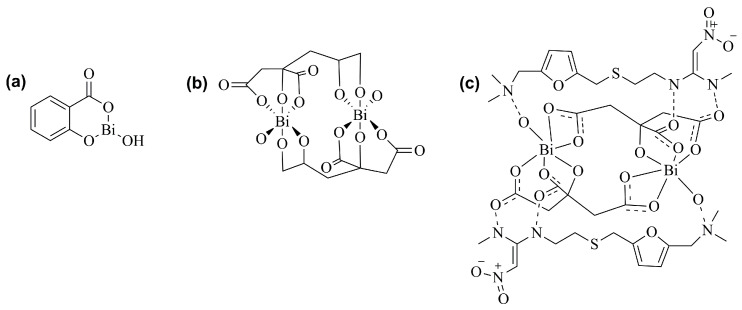
Chemical structures of (**a**) bismuth subsalicylate, (**b**) colloidal bismuth subcitrate, and (**c**) ranitidine bismuth citrate [35,36,37].

Nowadays, bismuth subsalicylate, colloidal bismuth subcitrate, and ranitidine bismuth citrate (Figure 2) [35,36,37] are still utilized to treat various gastrointestinal diseases worldwide, frequently associated with *Helicobacter pylori* infections [38,39,40,41,42].

*H. pylori*, a microaerophilic pathogen, is able to preclude ulcers from healing. In this context, bismuth derivatives can precipitate within the ulcer, giving rise to the development of a glycoprotein–bismuth complex that acts as a protective coating, contributing to the healing of the lesion [32]. In addition, due to the inhibition of the activity of this bacterium, bismuth compounds have an anti-ulcer activity. Interestingly, a low potential to the development of bacterial resistance to bismuth compounds was observed, which can be attributed to different mechanisms. These include, for example, the inhibition of urease UreG and of bacterial enzymes such as metallo-β-lactamases, due to the displacement of the Zn(II) cofactor [43]. Therefore, based on the effectiveness of bismuth compounds, such as bismuth subsalicylate, in the treatment of *H. pylori*, other novel compounds have also been developed for this pathology. For example, Pathak et al. synthesized new bismuth(III) hydroxamate complexes that exhibited a good activity against three strains of *H. pylori* [44]. In addition, other compounds containing this element, such as bismuth(III) 5-sulfosalicylate complexes, phenylbismuth(III) sulfonate complexes, bismuth subcarbonate nanoparticles (NPs), and bismuth–fluoroquinolone complexes have also showed activity against this bacterium [45,46,47,48].

At the clinical level, a trial aimed to investigate the effect of bismuth salts in the presence or absence of acid suppression. For this, *H. pylori*-positive patients were distributed in a control group, in a group receiving colloidal bismuth subcitrate at a dose of 125 mg/tab, and in a group taking colloidal bismuth subcitrate plus a high dose of the proton-pump inhibitor esomeprazole at a dose of 40 mg. The results showed that the acid suppression did not interfere in the activity of bismuth salts towards *H. pylori* [49].

**Figure 3:**
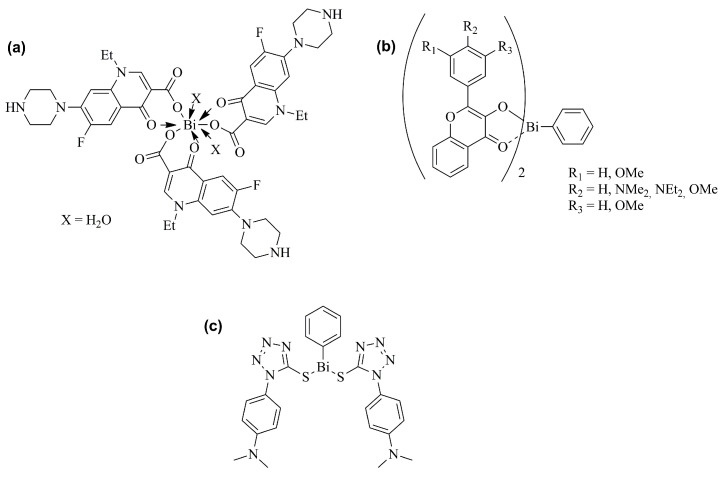
Examples of chemical structures of bismuth-containing compounds with antibacterial activity. (**a**) bismuth-norfloxacin complex; (**b**) homoleptic bismuth(III) tris-flavonolate complexes; (**c**) bismuth thiolates [50,52,58].

Regarding antibacterial effects in general, bismuth(III) flavonolates have demonstrated antibacterial activity either against Gram-positive (*Staphylococcus aureus*, methicillin-resistant *Staphylococcus aureus*, and vancomycin-resistant *Enterococcus*) and Gram-negative (*Escherichia coli*, *Pseudomonas aeruginosa*) bacteria [50]. Similar results were found with di-aryl bismuth phosphinates [51]. Regarding bismuth thiolates, their site of action was the cell membrane in methicillin-resistant *Staphylococcus aureus* [52]. In addition, bismuth NPs showed efficacy in reducing biofilm-forming bacteria in the oral cavity (minimum inhibitory concentration of 2.5 and 5 μg/mL against *Streptococcus salivarius* and *Enterococcus faecalis*, respectively) [53]. Other bismuth derivatives with antibacterial activity can also be found in the literature, such as cyclic organobismuth molecules [54], bismuth(III) phenyl pyrazolinates [55], bis(dialkyldithiocarbamato)diorganodithiophosphatobismuth(III) complexes [56], salicylate and pyrazoline complexes of bismuth(III) [57], and bismuth–norfloxacin complexes [58]. Examples of these compounds are illustrated in Figure 3. This panoply of compounds evaluated for this therapeutic indication effectively evidences the potential of bismuth derivatives as antibacterial agents.

**Figure 4:**
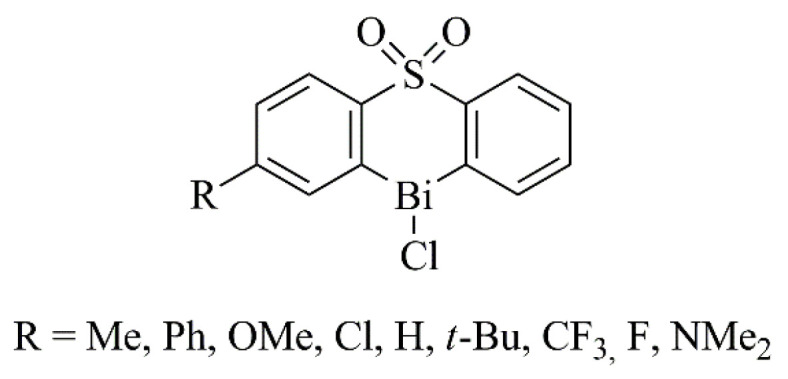
Chemical structure of heterocyclicorganobismuth(III) compounds derived from diphenyl sulfone with antifungal activity [62].

The antileishmanial and antifungal activity of bismuth compounds has also been explored [59]. An example is the work of Andleeb et al., who synthesized a set of heteroleptic triorganobismuth(V) biscarboxylates and found that some of them presented high activity against the promastigotes of *Leishmania tropica* [60]. Regarding antifungal properties, triarylbismuth dihalides and heterocyclicorganobismuth(III) compounds derived from diphenyl sulfones (Figure 4) displayed inhibitory action against *Saccharomyces cerevisiae*, probably due to the coordinated geometry of the bismuth atom [61,62].

**Figure 5:**
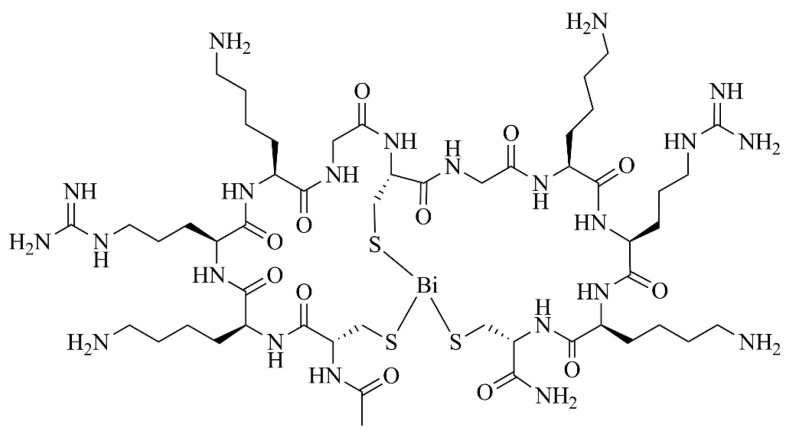
Chemical structure of a peptide–bismuth bicyclic derivative with antiviral activity [63].

Beyond the action against bacteria, fungi, and parasites, bismuth compounds have also been studied for the treatment of pathologies caused by viruses. In this context, peptide–bismuth bicycles (Figure 5) exhibited inhibition constants of 23 and 150 nM towards proteases from the Zika and West Nile viruses, respectively [63]. In addition, ranitidine bismuth citrate suppressed severe acute respiratory syndrome coronavirus 2 (SARS-CoV-2) replication, decreasing viral loads in both upper and lower respiratory tracts, and was able to relieve virus-associated pneumonia in a golden Syrian hamster model [35]. At the clinical level, the treatment with bismuth subsalicylate improved the clinical results of a COVID-19-positive Crohn’s disease patient [64].

### 2.2. Antitumor Effects

**Figure 6:**
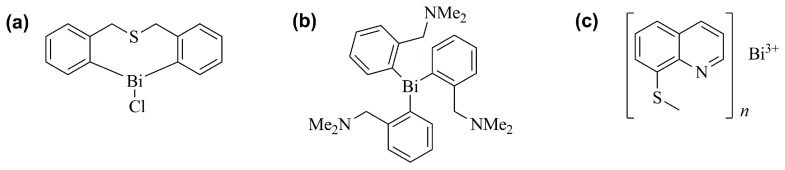
Examples of chemical structures of bismuth-containing compounds presenting anticancer activity. (**a**) bi-chlorodibenzo[*c*,*f*][1,5]thiabismocine, (**b**) tris[2-(*N*,*N*-dimethylaminomethyl)phenyl]-bismuthane, and (**c**) bismuth 8-quinolinethiolates [66,67,68].

Biocoordination studies of bismuth compounds suggest that their principal targets are non-DNA sites, offering a change for new directed approaches in the treatment of cancer [34]. Therefore, a number of research groups have prepared several synthetic bismuth molecules, including organo- and inorgano-bismuth derivatives, and evaluated their in vitro cytotoxic or antiproliferative activities against various cancer cell lines. Relevant antiproliferative effects were proven for several compounds, which in some cases were superior to those observed with classical anticancer agents, such as cisplatin [34]. In this context, these bismuth derivatives include heterocyclic organobismuth derivatives, tris[2-(*N*,*N*-dimethylaminomethyl)phenyl]-bismuthane, bismuth 8-quinolinethiolates, and bismuth NPs [65,66,67,68,69]. Examples of bismuth derivatives presenting anticancer activity are represented in Figure 6.

Some mechanisms of the antitumor action of bismuth compounds have been explored. In this context, bismuth(III) dithiocarbamate complexes induced intrinsic apoptotic pathways in MCF-7 cells, modulated several cancer related genes, and inhibited the NF-κB signaling pathway [70]. Increased phagocytic activity and DNA repair foci were also reported for RAW 264.7 cells exposed to bismuth NPs [71].

Also in the context of anticancer activity, Stoltenberg and collaborators [30] evaluated bismuth uptake by lysosomes of the J774 histiocytic lymphoma cell line. These cells were incubated with increased concentrations of bismuth citrate (5, 25, 100, and 200 µM) and evaluated at 6, 12, and 24 h. Taking into account the results, the authors suggested that concentrations higher than 5 µM reduced cells’ attachment, which was more evident with increasing exposure times. Moreover, the exposure during 12 or more hours at 100 µM or 6 h of incubation at 200 µM to bismuth citrate led to the disintegration of cell membranes [30]. The cytotoxicity of common bismuth compounds in human thyroid cancer cells was also investigated by Kobayashi et al. The compounds tested included bismuth acetate, bismuth chloride, bismuth subgallate, and bismuth subsalicylate. These authors found some cytotoxicity only in the cells treated with bismuth subgallate [72].

The in vitro cytotoxic effects of bismuth NPs was studied in HeLa and MG-63 cancer cells. It was found that the different types of bismuth NPs explored led to higher cytotoxicity in HeLa cells compared with MG-63 cells [73]. In addition, Song et al. evaluated the neurological toxicity of bismuth ferrite NPs in vitro using PC12 cells. The observed cytotoxicity was concentration-dependent, as the cells viability decreased from 95% to 73% with increasing concentrations from 10 to 200 μg/mL, after 3 h of incubation. The mechanism of action was investigated using the annexin V-FITC apoptosis detection kit, which demonstrated that the exposure of the cells to bismuth ferrite NPs led to only a small percentage of apoptotic (below 2%) and necrotic (below 10%) cells [74].

Other authors have evidenced that autophagy was involved in bismuth-NPs-induced toxicity in human embryonic kidney cells. In addition, it was also demonstrated that bismuth NPs are able to enter cells in a dose- and time-dependent manner [75]. Abudayyak et al. investigated the toxic effects of bismuth(III) oxide NPs in human hepatocarcinoma cells, human kidney epithelial cells, human colorectal adenocarcinoma cells, and human lung carcinoma cells. They observed that the main cell death pathways were apoptosis in the hepatocarcinoma and kidney cells, and necrosis in lung and colorectal cells [76].

Targeted radiation therapy is a known strategy for the treatment of cancer, being an approach mostly considered in inoperable tumors, tumors located close to radiation sensitive organs, metastatic disease, and diseases such as leukemia and lymphoma. This type of therapy involves the use of carrier molecules, for example, antibodies and peptides, specifically targeting cancer cells, and a selected radionuclide that without affecting surrounding healthy tissue should emit controlled doses of ionizing radiation to cancer cells [77,78]. A radionuclide’s half-life and the existence of viable chemistry are the most important variables that affect its use or viable supply [34]. The radionuclides ^212^Bi and ^213^Bi are probably the most investigated α-emitters in cancer therapy. Indeed, these radionuclides can be stably bound to diverse chelating agents, which can be conjugated to several structures, such as monoclonal antibodies, peptides, or other vectors. The in vivo stable sequestration of these radionuclides could also be considered to improve the delivery of radiation to tumors and increase their safety profile (e.g., renal toxicity). Therefore, several researchers have developed ^213^Bi-based systems to improve several parameters, such as the chelation and/or radiolabeling chemistry, targeting vectors, and radionuclide delivery [77,78,79].

Despite the therapeutic interest of ^213^Bi derivatives in cancer therapy, some drawbacks have been pointed out regarding the use of these compounds, namely their high costs, unresolved chemistry, and poor availability. Moreover, the in vivo stability and issues of metabolism are not well determined, as well as radiologic side effects [34].

In addition to their anticancer properties, several reports described the role of bismuth derivatives in decreasing the side effects of clinically available anticancer drugs. For example, it was proved that bismuth zinc citrate can reduce cisplatin-induced nephrotoxicity, probably due to the stimulation of the antioxidant protein metallothionein and glutathione conjugation [80,81].

### 2.3. Other Properties

In 2021, Brum et al. published a systematic review and meta-analysis of bismuth subsalicylate effectiveness in the prevention and treatment of diarrhea [82]. In fact, this drug has proven to be efficacious in both adults and children [82,83]. Moreover, a placebo-controlled randomized clinical trial demonstrated that patients with diarrhea who received bismuth subsalicylate have a reduced antibiotic consumption [84], which is an important finding concerning the actual problem of antibiotic resistance.

Interestingly, compounds containing bismuth have also been explored towards Alzheimer’s disease. This is the case of few-layer bismuth selenides previously exfoliated by hemin to potentiate their action. It was found that smaller and thinner compounds presented the highest inhibition of amyloid β (Aβ)_1–2_ aggregation. In addition, this activity was associated to the high adsorption capacity of few-layer bismuth selenides for Aβ_1–42_ monomers and to the decrease in Aβ-mediated peroxidase-like activity [85].

## 3. Bismuth Toxicity

With the continuous growing use of bismuth, it has become clear that this element is present in our environment and in our daily lives, which urges a more in-depth investigation into its possible toxic effects. The molecular mechanisms of bismuth toxicity remain unclear. However, it has been proposed to result from its binding to essential enzymes, the reduction of the cerebral blood flow, and lactate accumulation, thus interfering in the oxidative metabolism of the central nervous system, which leads to neurotoxicity [86,87]. Regarding nephrotoxicity, the renal tubular impairment caused by bismuth compounds can be associated with the inactivation of the sulphydryl groups important for active tubular transport processes [87].

In the following sections, the main data of bismuth toxicity in preclinical and clinical studies will be discussed.

### 3.1. Preclinical Studies

#### 3.1.1. In Vitro Studies

Several studies have been performed considering the cytotoxicity of bismuth compounds. These explored relatively common bismuth salts (e.g., bismuth citrate) and other derivatives, as well as bismuth-based nanoparticles.

An interesting study was carried out by Gao et al. [88], who used HaCaT keratinocytes (a non-cancer cell line). These authors showed that bismuth oxybromide induced a loss of cell viability in a concentration-dependent pattern. Through the annexin-V/propidium iodide flow cytometric analysis, these authors also observed that this bismuth derivative triggered late apoptosis. In addition, this compound provoked a loss of cell membrane integrity and, consequently, cell death. Further, the same research group demonstrated that microsphere-shaped bismuth oxychloride nanomaterials were associated with a lower toxicity compared to the nanosheet-shaped form, probably due to weaker particle–membrane interactions. The increased interactions with the cell membrane observed for microsphere-shaped NPs was related to the presence of surface hydroxyls [89].

The cellular uptake, the cytotoxicity, and genotoxicity of monomethylbismuth, bismuth citrate, and bismuth glutathione have been explored by Von Recklinghausen et al. [14] in HepG2 cells, and human lymphocytes and erythrocytes. The uptake of bismuth glutathione was relatively low (<0.3%) in all of the tested cells, whereas the uptake of bismuth citrate was 2.6% and 6.5% by lymphocytes and erythrocytes, respectively. Regarding methyl bismuth, its uptake was markedly high (up to 23% by lymphocytes and 36% by erythrocytes) [14]. The cytotoxic effects in hepatic cells were relevant after methyl bismuth treatment for 1 h at concentrations ≥ 350 μM and after 24 h at concentrations ≥ 130 μM. In erythrocytes, methyl bismuth was toxic at concentrations ≥ 3.8 μM (>50% cell death) after 24 h of exposure. On the other hand, methyl bismuth displayed cytotoxic effects in lymphocytes only at concentrations > 430 μM after 24 h of incubation. Bismuth citrate led to 48% of cell death in erythrocytes (concentrations ≥ 113 μM, 24 h). Regarding genotoxicity, methyl bismuth led to chromosomal type aberrations (single- and double-strand breaks) in lymphocytes. Overall, the authors concluded that the methylated bismuth compound was more membrane permeable and presented more cytotoxicity than other bismuth derivatives, such as bismuth glutathione and bismuth citrate [14]. In addition, the effects of trimethylbismuth in Caco-2, CHO-9, and HepG2 cancer cells were studied by Dopp and colleagues. The results demonstrated that this methylated derivative was cytotoxic in all of the tested cell lines (LC_50_: 110 μmol/Lgv for Caco-2, 128 μmol/Lgv for CHO-9, and 194 μmol/Lgv for HepG2) [90].

The toxicity of bismuth-based NPs was also explored by other research groups. For example, the genotoxicity of bismuth(III) oxide NPs was evaluated on the root cells of *Allium cepa* using Allium and Comet assays. Genotoxic effects were found through the increase in the mitotic index and in the DNA damage at the concentrations of 25, 50, 75, and 100 ppm [91].

#### 3.1.2. In Vivo Studies

Bismuth pellets attracted attention after the forbiddance of the use of lead in shotgun pellets. Therefore, Pamphlett and collaborators searched for other alternatives, such as bismuth compounds, to be released from shotgun pellets that had been inserted into mice, namely into the peritoneal cavity. Bismuth was found in the cerebrum, brain stem, spinal cord, posterior root ganglia, and renal tubular cells, and in the lungs, liver, and spleen [27]. Moreover, bismuth was also detected in the testis of rats exposed to bismuth subnitrate. In fact, bismuth traces were found in the interstitial tissue and in the seminiferous tubules. A higher amount was detected in Leydig cells [29]. The same research group showed that bismuth subnitrate can possibly access the nervous system through retrograde axonal transport in rats [28].

The gastrointestinal absorption and systemic uptake of bismuth compounds, such as bismuth citrate and ranitidine bismuth citrate, were also investigated after oral administration in mice. It was found that bismuth was absorbed in gastrointestinal epithelial cells as shown by bismuth staining in gastric, duodenal, and epithelial cells. Moreover, bismuth was only significantly present in lysosomes, and high bismuth concentrations led to cell signs of toxic degradation, including cytoplasmic vacuolation and intracellular swelling [92].

The acute and chronic (28-days) oral toxicity of elemental bismuth were investigated by Sano et al. [93] in rats and no abnormal clinical signs were found in either dose regimen. Therefore, the authors suggested a good safety profile for the elemental bismuth present. In addition, the effects of low, medium, and high doses of bismuth (0.8, 4 and 20 mg/kg) were evaluated after a 13-week intratracheal intermittent administration. No abnormal clinical signs were associated with bismuth administration in this study. However, hair loss was observed in three animals at medium and high doses of bismuth, as well as the prevention of body weight gain from Day 29 at the highest dose (not statistically significant). Slight alterations in hematological parameters were also observed, as well as brown patches in the lungs of animals of all dose groups, and black patches and lung collapses in all animals from the groups of the highest doses (4 and 20 mg/kg). The enlargement of bronchial lymph nodes and a white patch in the liver were also detected in animals of all groups [94].

The long-term toxicity of ^213^Bismuth-labelled bovine serum albumin (^213^Bi-BSA) was also investigated in NMRI-nu (nu/nu) mice. In this study, 3.7, 7.4, and 11.1 MBq of ^213^Bi-BSA were intravenously administered to the animals, which were then monitored by 55 weeks. It was found that mice died from liver and kidney failure when ^213^Bi-BSA was administered at the highest dose. Moreover, the liver toxicity was associated with an increase in alanine aminotransferase and aspartate aminotransferase levels. Regarding the group of mice that received ^213^Bi-BSA at 7.4 MBq, they presented an increase in plasma blood urea nitrogen and creatinine due to impaired kidney function. On the other hand, the injection of ^213^Bi-BSA at the lowest dose (3.7 MBq) revealed to be safe, without plasma enzyme modifications or histological abnormalities [95].

Omouri et al. described the bioavailability and chronic effects of bismuth in the earthworm *Eisenia andrei* exposed to soil artificially contaminated by bismuth citrate. The results indicated that bismuth decreased the reproduction parameters of *Eisenia andrei* at concentrations above 116 mg/kg [96]. However, it was also found that bismuth did not affect the growth and survival of this worm. Different results were found when bismuth−asparagine coordination polymer spheres were tested in zebrafish embryos. In this case, it was demonstrated that the spheres containing bismuth have the potential to cause developmental toxicity in a concentration-dependent pattern [97].

### 3.2. Clinical Evidences

In humans, it is known that exposure to excessive bismuth concentrations can lead principally to renal failure associated with degeneration and necrosis of the renal proximal tubules epithelium, liver necrosis, reversible dysfunction of the nervous system, pigmentation of the gums and intestine, and skin eruptions [98,99].

#### 3.2.1. Toxicity after Systemic Inadequate Use of Bismuth-Based Drugs

A summary of the clinical cases found in the literature was performed, aiming to understand bismuth toxicity in humans, particularly those of the inadequate use of bismuth-based products in systemic use (Table 1). 

The most notorious situation of bismuth toxicity in man is probably the French outbreak of bismuth encephalopathy. In this context, Supino-Viterbo and colleagues reported the cases of 45 patients with this condition, who were evaluated by electroencephalogram (EEG) studies. All of the patients in the study had been treated with bismuth subnitrate between 5 and 20 g/daily, over a period of 4 weeks to 30 years. The blood bismuth levels (Table 1, Entry 1) on the day of the EEG ranged between 150 and 1600 µg/L (the normal values are less than 20 µg/L). In urine samples, the levels of bismuth registered were from 200 to 9600 µg/L [25].

In other report, Hudson et al. described the case of a young man (27 years old) admitted into hospital 4 h after taking 100 De-Nol^TM^ tablets (total of 12 g of colloidal bismuth), paracetamol, and alcohol. After 10 days of discharge, the patient was admitted again into hospital, due to anorexia, nausea, vomiting, general malaise, blurring of vision, and poor urinary output, but without signs of encephalopathy. Through biochemical analysis, a blood bismuth level of 260 µg/L and a urine bismuth concentration of 120 µg/L were found (Table 1, Entry 2). The patient was diagnosed with neurotoxicity and renal failure induced by bismuth and started hemodialysis. Five days later, the renal function and the neurological signs were solved [100].

Another case reported was the overdose of a 76-year-old man that took 80 De-Nol^TM^ tablets 4 h prior to hospital admission [101]. In this case, a level of 1600 µg/L bismuth was detected in the blood (Table 1, Entry 3). The patient was started with ranitidine, antiacid, and magnesium sulfate enemas to solve gastrointestinal symptoms and dialyzed for 3 days. He developed acute abdominal pain with absent bowel sounds, and died 4 days later. Necropsy showed a perforated duodenal ulcer and “pale kidneys”, which contained bismuth (11 mg/g and 16 mg/g, respectively) [101].

Other case reports have been described in the literature, evidencing the ingestion of high quantities of bismuth salts (>5 g). In these cases, gastrointestinal, renal, and neurological injuries were the most reported. In this context, Playford et al. described a case of a 68-year-old man, who took the double of the recommended dose of De-Nol^TM^ (864 mg bismuth a day) for two months (Table 1, Entry 4). Several physiological alterations were found in this patient, such as cerebral dysfunction, incontinence, visual hallucinations, bilateral grasp reflexes, and ataxia. The loss of alpha rhythm and diffuse slow waves, which were consistent with metabolic encephalopathy, were found in the EEG. The condition was resolved after the administration of the metal chelator 2,3-dimercapto-1-propanesulfonic acid (DMPS) for 10 days [102].

An example of renal damage is tubular necrosis that was diagnosed in young adults after the ingestion of high doses of products containing bismuth. In fact, a 21-year-old man was admitted with this condition 3 h after the ingestion of 39 tablets of bismuth subcitrate (Table 1, Entry 5). A crystalloid infusion was prescribed, since the patient demonstrated epigastric pain, but without success. After the renal function has deteriorated, a renal biopsy showed moderate acute tubular necrosis. However, bismuth was not found in the biopsy specimen [103]. In another case, a 16-year-old girl presented nausea, vomiting, dizziness, and oliguria after having taken 10–15 tablets of tripotassium dicitrato bismuthane one week before hospital admission (Table 1, Entry 6). A renal biopsy also supported the diagnosis of acute tubular necrosis [104].

Interestingly, in other case, the symptoms of a 76-year-old woman were misinterpreted as Alzheimer’s disease. However, later it was found that these symptoms were associated to bismuth toxicity, since she had been ingesting around 4 g of bismuth daily (Table 1, Entry 7) [105].

An accidental intoxication of a 2-year-old boy with 28 De-Nol^TM^ tablets was described by Islek and collaborators [22]. Bismuth blood levels were determined on Day 10 (Table 1, Entry 8), being observed at a value of 739 µg/L. The patient recovered and was discharged on Day 20 after admission, presenting blood bismuth levels of 96 µg/L and 12 µg/L on Days 60 and 150, respectively [22].

Another case included a 22-year-old woman who attempted suicide by taking 5.4 g of colloidal bismuth subcitrate (Table 1, entry 9). The patient was treated with the chelating agent DMPS by the intravenous route and hemodialysis to eliminate bismuth [106]. Cengiz et al. described a situation of a 16-year-old girl with nausea, vomiting, and facial paresthesia, who attempted suicide 10 days earlier by ingesting 60 De-Nol^TM^ tablets. The medical examination showed no signs of encephalopathy and a slightly modified kidney function. As reported in Table 1 (Entry 10), the serum bismuth levels were 495 µg/L 2 days after hospital admission, and the patient started with hemodialysis therapy. The oral treatment with the metal-chelating agent penicillamine was also prescribed. Seven weeks later, her renal function had returned to normal, and the serum bismuth levels had dropped to 260 µg/L [23].

In another case, a 56-year-old woman with several days of psychomotor retardation, tremor of hands, lack of concentration, visual hallucinations, and postural instability was treated for irritable bowel syndrome, hypertension, hypothyroidism, and depression, without success An EEG on admission revealed moderate and nonspecific encephalopathy. A more in-depth evaluation of the situation allowed the detection that the patient had begun taking bismuth subsalicylate two months earlier to control diarrheal symptoms of a collagenous colitis. In addition, she had increased the use of the drug over the past few weeks. Thereafter, biochemical parameters showed that the bismuth levels in her blood and urine were 397.3 ng/mL and 292.5 ng/mL, respectively (Table 1, Entry 11) [107].

Other report described the case of a 21-year-old woman who took 20 tablets of colloidal bismuth subcitrate in a suicide attempt (Table 1, Entry 12). The treatment consisted in a gastric lavage and intravenous fluid therapy. Blood chemistry and urine sediment analyses suggested renal dysfunction, despite normal plasma glucose concentration. Therefore, patient was treated with the chelating agent DMPS and hemodialysis. In this case, 8 weeks after discharge, the patient’s renal function remained abnormal [24]. 

Another case of a suicidal attempt involved a 16-year-old girl who took 19 g of bismuth subcitrate potassium (De-Nol^TM^) (Table 1, Entry 13) [108]. In this situation, the patient developed acute renal failure and toxic metabolic encephalopathy. On the 20th day of hospital admission, the biochemical parameters of the patient began to normalize [108].

A case of a 50-year-old woman with a history of irritable bowel syndrome that arrived at the emergency department with disorientation, inattention, memory loss, and tremors was also reported. About four weeks later, when the patient began to improve and was to communicate, she revealed that she self-medicates with supplements bought online, one of those being bismuth subgallate, which she was taking 3 to 5 times a day (Table 1, Entry 14). There was no specific treatment administered as the patient continued to improve [109].

Disel et al. described a case of a 34-year-old woman admitted to a hospital with complaints of nausea and vomiting, two days after intentionally taking 8 De-NolTM tablets (2400 mg of bismuth subcitrate). She revealed apathy and blue-black discoloration in the teeth and gums (Table 1, Entry 15), and a gastric lavage was carried out. A complete urinary analysis showed proteinuria, glycosuria, and hemoglobinuria. She was then diagnosed with acute renal failure, probably due to bismuth toxicity, and was hospitalized in the critical care unit. Since bismuth is weakly bonded to plasma proteins, plasmapheresis was performed. On the 24th day of hospitalization, the patient was discharged [110].

A 44-year-old woman, who used bismuth subsalicylate for around 20 years, presented abnormal behavior and postural instability. Moreover, the examination of the patient also revealed a greyish discoloration of teeth, confusion, and generalized myoclonic jerks. Bismuth levels were found highly above of the threshold for toxicity in urine (375 μg/L), serum (260 μg/L), and cerebrospinal fluid (21.4 μg/L) (Table 1, Entry 16). Further, full recovery was achieved with supportive treatment and bismuth discontinuation [111]. Another report described the case of a 77-year-old woman, who was treated with bismuth subsalicylate for biopsy-proven collagenous colitis. After a marked decline in her cognitive and physical status, a high urinary bismuth level (2117 nmol/L) was found (Table 1, Entry 17). The discontinuation of the therapy led to the improvement of the symptoms [112]. A case of coagulopathy was also reported in a 62-year-old woman with underlying cirrhosis due to bismuth subsalicylate (Table 1, Entry 18) [113].

#### 3.2.2. Toxicity after Local Application of Bismuth Iodoform Paraffin Paste

Bismuth iodoform paraffin paste (BIPP) contains bismuth subnitrate and iodoform as active ingredients. BIPP has been used to pack cavities in ear, throat, nose, dental, and neurosurgical practice, acting as an antiseptic and astringent. However, some cases of toxicity associated with this bismuth-based product have also been described.

For instance, a case of a 57-year-old woman was reported by Sharma et al. Two months after the use of BIPP pack, the patient became confused and agitated with intermittent bihemispheric signs. After five months, the BIPP pack was discontinued, and the condition of the patient improved. When the BIPP pack was reapplied, the mental alterations returned and the possibility of bismuth toxicity was considered, with the BIPP pack being removed. At this time, the blood bismuth concentration was 52 ng/L (Table 2, Entry 1). Further, the patient’s conscious level was improved and the blood bismuth concentration reduced to almost half [114].

Another case included an 86-year-old woman admitted to the hospital for a partial maxillectomy (Table 2, entry 2). This patient underwent the surgery with split skin grafting to the maxillary antrum that was packed with BIPP. Five days after the surgery the patient presented as exhausted, lightheaded, and unsteady, and on Day 11 she was barely eating and having several fainting episodes. Three days later, the BIPP pack was removed and the patient was still confused and aggressive, but one week after the pack removal, she began to improve and become cooperative [115].

Roest et al. reported three cases of otitis externa caused by allergic contact associated with BIPP (Table 2, Entry 3) [116]. All these cases included women with external auditory meatus and concha, which were packed with BIPP-impregnated gauze following surgery.

An 81-year-old man presented confusion, dysphagia, and incontinency two days after a surgical procedure. It was found that a nasal packing with BIPP was used when prolonged packing with Merocel^TM^ failed to stop the epistaxis. The patient’s serum bismuth level was 250 µg/L, as reported in Table 2 (Entry 4), and the bismuth toxicity was associated with the patient’s state of confusion [117].

In another case, a 67-year-old man with a sacral chondroma was surgically resected and packed with gauze soaked in BIPP. Five days after the surgical procedure, the patient was confused, disorientated, delusional, and aggressive. Moreover, he reported abdominal discomfort, nausea, and tremor. By Day 10, the patient’s condition deteriorated and bismuth toxicity was considered due to the fact that the patient developed myoclonic jerks with intermittent episodes of drowsiness and worsening confusion. It was found that his blood and urine concentrations of bismuth were 340 µg/L and 2800 µg/L, respectively (Table 2, Entry 5). Therefore, the BIPP packing was removed and the patient was treated with intravenous chelation therapy with DMPS. The patient’s condition markedly improved, and blood and urine bismuth levels declined [118].

Two additional cases of reactions to BIPP packs were described by Atwal et al. [119] (Table 2, Entry 6). In the first case, a 59-year-old man was packed with BIPP-impregnated gauze, due to a keratocystic odontogenic tumor. This patient presented as fatigued, confused, apathetic, forgetful, and suffered spasms. Biochemical analysis showed a blood bismuth concentration of 109.9 nmol/L, which led to BIPP removal. After 18 months, the blood bismuth concentration dropped to 0.02 nmol/L. The second case included a 92-year-old-woman who had a BIPP pack placed after a hemimaxillectomy. Nine days after the surgical procedure, she became progressively confused. The concentration of her blood bismuth concentration was 144.0 nmol/L. Then, the BIPP pack was removed and her condition gradually improved. About 4 months after the pack removal, the blood bismuth concentration was 8.9 nmol/L [119].

The case of a 72-year-old female who underwent a sinonasal and orbital debridement of infected tissue, and later an orbital exenteration with partial maxillectomy, was also reported. Her exenteration cavity was packed with 6 BIPP impregnated gauzes. After developing suspect symptomatology, the serum bismuth level was determined, being 391 nmol/L (Table 2, Entry 7). The removal of the BIPP pack led to the resolution of her symptoms, and four months later, her serum bismuth concentration was 120 nmol/L [120]. Another report involved an elderly patient with BIPP-induced encephalopathy. In this case, the toxicity was associated with several factors, such as the high extension of the wound, the amount of BIPP packing, and the impaired renal function. After the removal of the BIPP packs, the patient gradually improved over the next two weeks without further intervention (Table 2, Entry 8) [121].

## 4. Conclusions

Bismuth compounds have been extensively used in clinical practice, mainly for the treatment of gastrointestinal disorders, such as gastric ulcers, dyspepsia, and H. pylori infections. Moreover, due to their interesting profile in terms of costs, safety, and biological properties, they have been studied for application in other infections for which they are not approved, as well as for cancer therapy.

Despite a significant number of case studies reporting bismuth-associated undesired effects, the toxicity of bismuth-based products and the associated mechanisms have not yet been fully understood. However, mainly due to the referred advantages, bismuth continues to be used in drug formulations. Since the information on this subject is relatively scarce, an effort must be made to better understand the bismuth safety profile. For this objective, additional studies should be performed, including in vitro and in vivo assays. In fact, in vitro studies at the cellular level should be performed, particularly involving relevant cell lines and several different bismuth compounds used today, either in drug synthesis or as active pharmaceutical ingredients. In addition, in vivo studies are of major importance, since animal models present complex biological interactions and physiological features more closely related to humans [122]. It is also important to mention the necessity to analyze immunological effects, as well as long-term systemic safety.

Regarding the clinical trial data, the most described adverse effects associated with bismuth compounds are acute neurotoxic and nephrotoxic effects in situations of overuse. However, the majority of the cases reported the reversibility of these effects, being resolved with the bismuth-based therapy discontinuation. Interestingly, other studies report the absence of serious neurological symptoms in patients receiving an association of bismuth subcitrate, metronidazole, and tetracycline for the eradication of *H. pylori* [123]. Moreover, as reported herein, bismuth compounds are able to reduce the toxic adverse effects of chemotherapeutic drugs, such as cisplatin, and they are important weapons as antibacterial agents, since they can contribute to the reduction of pharmacoresistance.

Overall, the existing data suggest that bismuth-based products are relatively safe and should continue being used, namely at the clinical level, given their important advantages. However, their toxicity profile has not yet been fully understood and more studies are needed, not only at the molecular and cellular level, but also in clinical settings with a focus on evaluating long-term systemic safety.

## Figures and Tables

**Table 1:** Reported cases of overdose of bismuth compounds.

Entry	Gender (Age [Years])	Quantity Consumed	Time from Ingestion to Hospitalization	Symptoms	Bismuth Concentration before Therapy	Bismuth Concentration after Therapy	Main Findings	Treatment	**References**
1	M/F (24–80)	5–20 g bismuth subnitrate daily	4 weeks–30 years	Depression, anxiety, irritability, delusions, phobias, somnolence, hallucinations, anorexia, sleep disorder, motor incoordination, jerky movements	Blood: 150–1600 µg/L Urine: 200–9600 µg/L	-	Monomorphic waves at 3 to 5 Hz; diffuse beta rhythm of low voltage	-	[25]
2	M (27)	100 De-Nol^®^ Tablets (12 g colloidal bismuth)	10 days	Anorexia, vomiting, nausea, legs weakness, blurring of vision, thirst, poor urinary output	Blood: 260 µg/L Urine: 120 µg/L Stools: 26.9 mg/g	96 days after ingestion: Blood: 8 µg/g	Opacification of the colon; non-specific slow-wave changes to both hemispheres	Purgation (magnesium sulfate), rehydration, hemodialysis	[100]
3	M (76)	80 De-Nol^®^ Tablets	4 h	Confusion, epigastric tenderness	Blood: 1600 µg/L	-	Opacification of the colon; acute tubular necrosis	Ranitidine, antacid, magnesium sulfate enemas, dialysis (3 days)	[101]
4	M (68)	Twice the recommended dose of DeNol^®^ (864 mg daily) for 2 months	-	Cerebral dysfunction, incontinence, bilateral grasps reflexes, hallucinations, ataxia	Blood: 880 µg/L Urine: 230 µg/L	-	Loss of alpha rhythm and diffuse slow waves consistent with a metabolic encephalopathy	Heavy-metal chelator 2–3 dimercapto-1 propane sulphonic acid (DMPS)	[102]
5	M (21)	39 tablets of bismuth subcitrate	-	Epigastric pain	Blood: ~200 µg/L Serum: ~1500 µg/L	Blood: ~125 Serum: ~10	Acute tubular necrosis	Intravenous furosemide, dopamine, mannitol, crystalloids	[103]
6	F (16)	10–15 tablets of tripotassium dicitrato bismuthane	1 week	Nausea, vomiting, dizziness, oliguria	-	-	Acute tubular necrosis	Hemodialysis, protein restriction, metoclopramide, aluminum hydroxide	[104]
7	F (76)	Pepto-Bismol^®^ (4.14 mg daily for 7 years)	-	Confusion, poor appetite, disturbed sleep, muscle twitching	On day 6: Serum—242 µg/L	After 30 days: Serum: 90 µg/L After 76 days: Serum: 14 µg/L	Normal X-ray; moderate atrophy; ventricular enlargement; ischemic white matter disease	Penicillamine, oral fluids, salt tablets, Cognex (Tacrine)	[105]
8	M (2)	28 De-Nol^®^ tablets (8.4 g of colloidal bismuth subcitrate)	6 h	-	On day 10: Blood: 739 µg/L Urine: 693 µg/L	Day 105: Blood: 12 µg/L	Opacification of the intestine and colon; normal magnetic resonance imaging (MRI)	Gastric lavage, intravenous saline, mannitol, furosemide	[22]
9	F (22)	5.4 g of colloidal bismuth subcitrate	2 h	-	Day 3: Serum: 640 µg/L	Day 11: Serum: 12 µg/L	Enlarged and edematous kidneys with thinning of the cortical area	DMPS, hemodialysis, hemodiafiltrations	[106]
10	F (16)	60 De-Nol^®^ tablets	10 days	Nausea, vomiting, facial paresthesia	Day 12: Serum: 495 µg/L	Day 64: Serum: 260 µg/L	Normal MRI	Hemodialysis, penicillamine	[23]
11	F (56)	45 mL (thrice per day) of bismuth subsalicylate (262 mg/15 mL)	-	Psychomotor retardation, decreased concentration, tremor of the hands, visual hallucinations, postural instability	Blood: 397.3 ng/mL Urine: 292.5 ng/mL	-	Moderate but nonspecific encephalopathy	Medication was held (bismuth subsalicylate)	[107]
12	F (21)	20 colloidal bismuth subcitrate tablets (300 mg of colloidal bismuth subcitrate)	4 h	-	-	-	Normal X-ray and MRI	Gastric lavage, intravenous fluids, DMPS, hemodialysis	[24]
13	F (16)	19 g of De-Nol^®^	1 h	-	-	-	Opacities in the left side of abdomen; intermittent rhythmic waves in the frontal region; hyper-intense signal alterations at bilateral parietal vertices of both cerebellar hemispheres	-	[108]
14	F (50)	200 mg of bismuth subgallate, 3 to 5 times a day	-	Disorientation, inattention, memory loss, tremors, myoclonic jerks, hyperreflexic with bilateral ankle clonus.	Serum: 44.4 µg/L Urine: 57.8 µg/L	-	Excessive theta activity	No specific treatment. Patient continued to improve	[109]
15	F (34)	8 De-Nol^®^ tablets (2400 mg bismuth citrate)	2 days	Nausea, vomiting, apathy, blue-black discoloration in the teeth and gums, proteinuria, glucosuria, hemoglobinuria	-	-	-	Plasmapheresis	[110]
16	F (44)	Pepto-Bismol^®^ (tablets of 150 mg bismuth subsalicylate)	20 years	Greyish discoloration of teeth, confusion, generalized myoclonic jerks, which worsened, reduction in alertness	Eight days after admission: Urine: 375 μg/L Serum: 260 μg/L Cerebrospinal fluid: 21.4 μg/L	One month after admission: Urine: 33 μg/L Serum: 13.1 μg/L	Diffuse and nonspecific cerebral dysfunction; no abnormalities reported	Supportive treatment	[111]
17	F (77)	Pepto-Bismol^®^ (bismuth subsalicylate 262.5 mg) one tablet three times daily	Around 1 year	Falls, tremors	Urine: 2117 nmol/L	-	-	No specific treatment	[112]
18	F (62)	Pepto-Bismol^®^ (half bottle per day)	5 days	1 week of watery non-bloody diarrhea and confusion	Blood: 4 µg/L Urine: 147.6 µg/L	-	-	Intravenous sodium bicarbonate, *N*-acetylcysteine infusions, one unit of fresh frozen plasma, two doses of 10 mg vitamin K intravenous	[113]

**Table 2:** Reported cases of bismuth iodoform paraffin paste (BIPP) toxicity.

Entry	Gender (Age [Years])	Surgery	Symptoms after Packing with BIPP	Bismuth Levels	Observations	References
1	F (57)	Removal of a basal cell carcinoma	Agitation, confusion, restlessness	52 ng/L	-	[114]
2	F (86)	Partial maxillectomy	Exhaustion, lightheadedness, poor appetite, tremor	Day 14: 146 nmol/L Day 22: 81 nmol/L	-	[115]
3	F (16)	Myringoplasty	Mild erythema and swelling of the concha	-	Allergic contact otitis externa due to BIPP	[116]
	F (13)	Myringoplasty	-	-	Allergic contact otitis externa due to BIPP	
	F (52)	Myringoplasty	Florid eczematous reaction	-	Allergic contact otitis externa due to BIPP	
4	M (81)	Epistaxis treatment with BIPP packing	Acute confusion, dysphagia	250 µg/L	-	[117]
5	M (67)	Resection of a sacral chondroma	Acute confusion, disorientation, delusions, aggressive, abdominal discomfort, nausea, tremor	Blood: 240 µg/L Urine: 2800 µg/L	-	[118]
6	M (59)	Marsupialisation and packing with BIPP of a keratocystic odontogenic tumor	Fatigue, confusion, apathy, forgetfulness, and spasms in the quadriceps	Blood: 109.9 nmol/L	After 18 months, blood bismuth concentration was 0.02 nmol/L	[119]
	F (92)	Right hemimaxillectomy	Confusion	Blood: 144.0 nmol/L	After 4 months, blood bismuth concentration was 8.9 nmol/L	
7	F (72)	Partial maxillectomy	Confusion, depressed mood, disorientation, aggression, behavioral change	Serum: 391 nmol/L	After 4 months, the serum bismuth concentration was 120 nmol/L	[120]
8	M (74)	Endoscopic nasopharyngectomy	Agitation, drowsiness, negative myoclonus	Day 7: Urine: 37,094 µg/L	Day 26—Urine bismuth levels: 457 µg/L Blood bismuth levels were not obtained in the early postoperative period, but were normal (13.6 ng/L) on Day 18	[121]

## Data Availability

Data are contained within the article or Supplementary Materials.

## Supplementary Materials

The following supporting information can be downloaded at: https://www.mdpi.com/article/10.3390/ijms25031600/s1. References [30,35,44,45,46,47,48,50,51,52,53,54,55,56,57,58,60,61,62,63,65,66,67,68,69,70,72,73,74,75,85] are cited in the supplementary materials.

## References

[1] Mohan R. (2010). Green Bismuth. Nat. Chem..

[2] Silvestru C., Breunig H.J., Althaus H. (1999). Structural Chemistry of Bismuth Compounds. I. Organobismuth Derivatives. Chem. Rev..

[3] Suzuki H., Ogawa T. (2001). Organobismuth(III) Compounds. Organobismuth Chemistry.

[4] Matias M., Campos G., Santos A.O., Falcão A., Silvestre S., Alves G. (2016). Potential Antitumoral 3,4-Dihydropyrimidin-2-(1H)-Ones: Synthesis, in Vitro Biological Evaluation and QSAR Studies. RSC Adv..

[5] Salvador J.A.R., Moreira V.M., Pinto R.M.A., Leal A.S., Le Roux C. (2011). Bismuth(III) Triflate-Based Catalytic Direct Opening of Oleanolic Hydroxy-γ-Lactones to Afford 12-Oxo-28-Carboxylic Acids. Adv. Synth. Catal.

[6] Pinto R.M.A., Salvador J.A.R., Le Roux C., Paixão J.A. (2009). Bismuth(III) Triflate-Catalyzed Direct Conversion of Corticosteroids into Highly Functionalized 17-Ketosteroids by Cleavage of the C17-Dihydroxyacetone Side Chain. J. Org. Chem..

[7] Pinto R.M.A., Salvador J.A.R., Le Roux C., Carvalho R.A., Beja A.M., Paixão J.A. (2009). Bismuth(III) Triflate-Catalyzed Rearrangement of 16α,17α-Epoxy-20-Oxosteroids. Synthesis and Structural Elucidation of New 16α-Substituted 17α-Alkyl-17β-Methyl-Δ13-18-Norsteroids. Tetrahedron.

[8] Salvador J.A.R., Silvestre S.M. (2005). Bismuth-Catalyzed Allylic Oxidation Using t-Butyl Hydroperoxide. Tetrahedron Lett..

[9] Matias M., Campos G., Silvestre S., Falcão A., Alves G. (2017). Early Preclinical Evaluation of Dihydropyrimidin(Thi)Ones as Potential Anticonvulsant Drug Candidates. Eur. J. Pharm. Sci..

[10] Gorbach S.L. (1990). Bismuth Therapy in Gastrointestinal Diseases. Gastroenterology.

[11] Wagstaff A.J., Benfield P., Monk J.P. (1988). Colloidal Bismuth Subcitrate. A Review of Its Pharmacodynamic and Pharmacokinetic Properties, and Its Therapeutic Use in Peptic Ulcer Disease. Drugs.

[12] Oliver T.E., Piantavigna S., Andrews P.C., Holt S.A., Dillon C.T. (2021). Interactions of Non-Steroidal Anti-Inflammatory Drugs and Their Bismuth Analogues (BiNSAIDs) with Biological Membrane Mimics at Physiological PH. Langmuir.

[13] Li H., Sun H. (2012). Recent Advances in Bioinorganic Chemistry of Bismuth. Curr. Opin. Chem. Biol..

[14] Von Recklinghausen U., Hartmann L.M., Rabieh S., Hippler J., Hirner A.V., Rettenmeier A.W., Dopp E. (2008). Methylated Bismuth, but Not Bismuth Citrate or Bismuth Glutathione, Induces Cyto- and Genotoxic Effects in Human Cells in Vitro. Chem. Res. Toxicol..

[15] Wang R., Li H., Sun H., Nriagu J.B.T.-E. (2019). Bismuth: Environmental Pollution and Health Effects. Encyclopedia of Environmental Health.

[16] Griffith D.M., Li H., Werrett M.V., Andrews P.C., Sun H. (2021). Medicinal Chemistry and Biomedical Applications of Bismuth-Based Compounds and Nanoparticles. Chem. Soc. Rev..

[17] Badrigilan S., Heydarpanahi F., Choupani J., Jaymand M., Samadian H., Hoseini-Ghahfarokhi M., Webster T.J., Tayebi L. (2020). A Review on the Biodistribution, Pharmacokinetics and Toxicity of Bismuth-Based Nanomaterials. Int. J. Nanomed..

[18] Rao R.B., Hoffman R.S., Howland M.A., Lewin N.A., Nelson L.S., Goldfrank L.R. (2015). Bismuth. Goldfrank’s Toxicologic Emergencies.

[19] Xin Y., Wang Z., Yao C., Shen H., Miao Y. (2022). Bismuth, a Previously Less-Studied Element, Is Bursting into New Hotspots. ChemistrySelect.

[20] Lopez E., Thorp S.C., Mohan R.S. (2022). Bismuth(III) Compounds as Catalysts in Organic Synthesis: A Mini Review. Polyhedron.

[21] Wang R., Li H., Ip T.K.-Y., Sun H., Sadler P.J., van Eldik R. (2020). Chapter Six—Bismuth Drugs as Antimicrobial Agents. Advances in Inorganic Chemistry.

[22] Işlek I., Uysal S., Gök F., Dündaröz R., Küçüködük Ş. (2001). Reversible Nephrotoxicity after Overdose of Colloidal Bismuth Subcitrate. Pediatr. Nephrol..

[23] Cengiz N., Uslu Y., Gök F., Anarat A. (2005). Acute Renal Failure after Overdose of Colloidal Bismuth Subcitrate. Pediatr. Nephrol..

[24] Erden A., Karahan S., Bulut K., Basak M., Aslan T., Cetinkaya A., Karagoz H., Avci D. (2013). A Case of Bismuth Intoxication with Irreversible Renal Damage. Int. J. Nephrol. Renovasc. Dis..

[25] Supino-Viterbo V., Sicard C., Risvegliato M., Rancurel G., Buge A. (1977). Toxic Encephalopathy Due to Ingestion of Bismuth Salts: Clinical and EEG Studies of 45 Patients. J. Neurol. Neurosurg. Psychiatry.

[26] Stoltenberg M., Danscher G. (2000). Histochemical Differentiation of Autometallographically Traceable Metals (Au, Ag, Hg, Bi, Zn): Protocols for Chemical Removal of Separate Autometallographic Metal Clusters in Epon Sections. Histochem. J..

[27] Pamphlett R., Danscher G., Rungby J., Stoltenberg M. (2000). Tissue Uptake of Bismuth from Shotgun Pellets. Environ. Res..

[28] Stoltenberg M., Schiønning J.D., Danscher G. (2001). Retrograde Axonal Transport of Bismuth: An Autometallographic Study. Acta Neuropathol..

[29] Stoltenberg M., Danscher G., Pamphlett R., Christensen M.M., Rungby J. (2000). Histochemical Tracing of Bismuth in Testis from Rats Exposed Intraperitoneally to Bismuth Subnitrate. Reprod. Toxicol..

[30] Stoltenberg M., Larsen A., Zhao M., Danscher G., Brunk U.T. (2002). Bismuth-Induced Lysosomal Rupture in J774 Cells. Apmis.

[31] Ge R., Sun H. (2007). Bioinorganic Chemistry of Bismuth and Antimony: Target Sites of Metallodrugs. Acc. Chem. Res..

[32] Sun H., Sun H. (2011). Biological Chemistry of Arsenic, Antimony and Bismuth.

[33] Rosário J., Moreira F., Rosa L., Guerra W., Silva-Caldeira P. (2023). Biological Activities of Bismuth Compounds: An Overview of the New Findings and the Old Challenges Not Yet Overcome. Molecules.

[34] Salvador J.A.R., Figueiredo S.A.C., Pinto R.M.A., Silvestre S.M. (2012). Bismuth Compounds in Medicinal Chemistry. Future Med. Chem..

[35] Yuan S., Wang R., Chan J.F.W., Zhang A.J., Cheng T., Chik K.K.H., Ye Z.W., Wang S., Lee A.C.Y., Jin L. (2020). Metallodrug Ranitidine Bismuth Citrate Suppresses SARS-CoV-2 Replication and Relieves Virus-Associated Pneumonia in Syrian Hamsters. Nat. Microbiol..

[36] Sandha G.S., Leblanc R., Veldhuyzen Van Zanten S.J.O., Sitland T.D., Agocs L., Burford N., Best L., Mahoney D., Hoffman P., Leddin D.J. (1998). Chemical Structure of Bismuth Compounds Determines Their Gastric Ulcer Healing Efficacy and Anti-*Helicobacter pylori* Activity. Dig. Dis. Sci..

[37] Ortiz-Aldaco M.G., Baéz J.E., Jiménez-Halla J.O.C. (2020). Bismuth Subsalicylate, a Low-Toxicity Catalyst for the Ring-Opening Polymerization (ROP) of l-Lactide (l-LA) with Aliphatic Diol Initiators: Synthesis, Characterization, and Mechanism of Initiation. RSC Adv..

[38] Yang N., Sun H. (2007). Biocoordination Chemistry of Bismuth: Recent Advances. Coord. Chem. Rev..

[39] Yoon J.Y., Kwak M.S., Jeon J.W., Cha J.M. (2021). Pretreatment with Ranitidine Bismuth Citrate May Improve Success Rates of *Helicobacter pylori* Eradication: A Prospective, Randomized, Controlled and Open-Label Study. Tohoku J. Exp. Med..

[40] Malfertheiner P., Bazzoli F., Delchier J.C., Celiñski K., Giguère M., Rivière M., Mégraud F. (2011). *Helicobacter pylori* Eradication with a Capsule Containing Bismuth Subcitrate Potassium, Metronidazole, and Tetracycline given with Omeprazole versus Clarithromycin-Based Triple Therapy: A Randomised, Open-Label, Non-Inferiority, Phase 3 Trial. Lancet.

[41] Tsang C.N., Ho K.S., Sun H., Chan W.T. (2011). Tracking Bismuth Antiulcer Drug Uptake in Single *Helicobacter pylori* Cells. J. Am. Chem. Soc..

[42] Chey W.D., Wong B.C.Y. (2007). Practice Parameters Committee of the American College of Gastroenterology American College of Gastroenterology Guideline on the Management of *Helicobacter pylori* Infection. Am. J. Gastroenterol..

[43] Li H., Wang R., Sun H. (2019). Systems Approaches for Unveiling the Mechanism of Action of Bismuth Drugs: New Medicinal Applications beyond *Helicobacter pylori* Infection. Acc. Chem. Res..

[44] Pathak A., Blair V.L., Ferrero R.L., Junk P.C., Tabor R.F., Andrews P.C. (2015). Synthesis and Structural Characterisation of Bismuth(III) Hydroxamates and Their Activity against *Helicobacter pylori*. Dalton Trans..

[45] Chen R., Cheng G., So M.H., Wu J., Lu Z., Che C.M., Sun H. (2010). Bismuth Subcarbonate Nanoparticles Fabricated by Water-in-Oil Microemulsion-Assisted Hydrothermal Process Exhibit Anti-*Helicobacter pylori* Properties. Mater. Res. Bull..

[46] Shaikh A.R., Giridhar R., Megraud F., Yadav M.R. (2009). Metalloantibiotics: Synthesis, Characterization and Antimicrobial Evaluation of Bismuth-Fluoroquinolone Complexes against *Helicobacter pylori*. Acta Pharm..

[47] Andrews P.C., Deacon G.B., Ferrero R.L., Junk P.C., Karrar A., Kumar I., MacLellan J.G. (2009). Bismuth(III) 5-Sulfosalicylate Complexes: Structure, Solubility and Activity against *Helicobacter pylori*. Dalton Trans..

[48] Andrews P.C., Busse M., Deacon G.B., Ferrero R.L., Junk P.C., Huynh K.K., Kumar I., Maclellan J.G. (2010). Structural and Solution Studies of Phenylbismuth(III) Sulfonate Complexes and Their Activity against *Helicobacter pylori*. Dalton Trans..

[49] Chiang T.H., Chen C.C., Tseng P.H., Liou J.M., Wu M.S., Shun C.T., Lee Y.C., Graham D.Y. (2021). Bismuth Salts with versus without Acid Suppression for *Helicobacter pylori* Infection: A Transmission Electron Microscope Study. Helicobacter.

[50] Burke K.J., Stephens L.J., Werrett M.V., Andrews P.C. (2020). Bismuth(III) Flavonolates: The Impact of Structural Diversity on Antibacterial Activity, Mammalian Cell Viability and Cellular Uptake. Chem. A Eur. J..

[51] Herdman M.E., Werrett M.V., Andrews P.C. (2022). Aryl Bismuth Phosphinates [BiAr2(O(O)PRR′)]: Structure–Activity Relationships for Antibacterial Activity and Cytotoxicity. Dalton Trans..

[52] Stephens L.J., Munuganti S., Duffin R.N., Werrett M.V., Andrews P.C. (2020). Is Bismuth Really the “Green” Metal? Exploring the Antimicrobial Activity and Cytotoxicity of Organobismuth Thiolate Complexes. Inorg. Chem..

[53] Rostamifar S., Azad A., Bazrafkan A., Modaresi F., Atashpour S., Jahromi Z.K. (2021). New Strategy of Reducing Biofilm Forming Bacteria in Oral Cavity by Bismuth Nanoparticles. BioMed Res. Int..

[54] Kotani T., Nagai D., Asahi K., Suzuki H., Yamao F., Kataoka N., Yagura T. (2005). Antibacterial Properties of Some Cyclic Organobismuth (III) Compounds. Antimicrob. Agents Chemother..

[55] Tripathi U.N., Siddiqui A., Solanki J.S. (2009). Synthesis, Spectral Characterization, and Antimicrobial Activity of Arsenic(III) and Bismuth(III) Phenyl)Pyrazolinates]. Turk. J. Chem..

[56] Chauhan H.P.S., Shaik N.M., Singh U.P. (2005). Synthetic, Spectroscopic and Antimicrobial Studies of Bis(Dialkyldithiocarbamato)Diorganodithiophosphatobismuth(III) Complexes. Appl. Organomet. Chem..

[57] Solanki J.S., Thapak T.R., Bhardwaj A. (2011). Synthesis, Structural Characterization, and in Vitro Antimicrobial Properties of Salicylate and Pyrazoline Complexes of Bismuth(III). J. Coord. Chem..

[58] Shaikh A.R., Giridhar R., Yadav M.R. (2007). Bismuth-Norfloxacin Complex: Synthesis, Physicochemical and Antimicrobial Evaluation. Int. J. Pharm..

[59] Andrews P.C., Frank R., Junk P.C., Kedzierski L., Kumar I., MacLellan J.G. (2011). Anti-Leishmanial Activity of Homo- and Heteroleptic Bismuth(III) Carboxylates. J. Inorg. Biochem..

[60] Andleeb S., Imtiaz-ud-Din, Rauf M.K., Azam S.S., Haq I.L., Tahir M.N., Zaman N. (2022). Structural Characterization and Antileishmanial Activity of Newly Synthesized Organo-Bismuth(V) Carboxylates: Experimental and Molecular Docking Studies. J. Biol. Inorg. Chem..

[61] Murafuji T., Miyoshi Y., Ishibashi M., Rahman A.F.M.M., Sugihara Y., Miyakawa I., Uno H. (2004). Antifungal Activity of Organobismuth Compounds against the Yeast *Saccharomyces cerevisiae*: Structure-Activity Relationship. J. Inorg. Biochem..

[62] Murafuji T., Fujiwara Y., Yoshimatsu D., Miyakawa I., Migita K., Mikata Y. (2011). Bismuth Heterocycles Based on a Diphenyl Sulfone Scaffold: Synthesis and Substituent Effect on the Antifungal Activity against *Saccharomyces cerevisiae*. Eur. J. Med. Chem..

[63] Voss S., Rademann J., Nitsche C. (2022). Peptide–Bismuth Bicycles: In Situ Access to Stable Constrained Peptides with Superior Bioactivity. Angew. Chem. Int. Ed..

[64] Wolf D.C., Wolf C.H., Rubin D.T. (2020). Temporal Improvement of a COVID-19-Positive Crohn’s Disease Patient Treated with Bismuth Subsalicylate. Am. J. Gastroenterol..

[65] Shakibaie M., Forootanfar H., Ameri A., Adeli-Sardou M., Jafari M., Rahimi H.R. (2018). Cytotoxicity of Biologically Synthesised Bismuth Nanoparticles against HT-29 Cell Line. IET Nanobiotechnol..

[66] Iuchi K., Tasaki Y., Shirai S., Hisatomi H. (2020). Upregulation of Nuclear Factor (Erythroid-Derived 2)-like 2 Protein Level in the Human Colorectal Adenocarcinoma Cell Line DLD-1 by a Heterocyclic Organobismuth(III) Compound: Effect of Organobismuth(III) Compound on NRF2 Signaling. Biomed. Pharmacother..

[67] Fujiwara Y., Mitani M., Yasuike S., Kurita J., Kaji T. (2005). An Organobismuth Compound That Exhibits Selective Cytotoxicity to Vascular Endothelial Cells in Vitro. J. Health Sci..

[68] Lukevics E., Shestakova I., Domracheva I., Nesterova A., Zaruma D., Ashaks J. (2006). Cytotoxicity of Metal 8-Quinolinethiolates. Chem. Heterocycl. Compd..

[69] García-Cuellar C.M., Cabral-Romero C., Hernández-Delgadillo R., Solis-Soto J.M., Meester I., Sánchez-Pérez Y., Nakagoshi-Cepeda S.E., Pineda-Aguilar N., Sánchez-Nájera R.I., Nakagoshi-Cepeda M.A.A. (2022). Bismuth Lipophilic Nanoparticles (BisBAL NP) Inhibit the Growth of Tumor Cells in a Mouse Melanoma Model. Anticancer. Agents Med. Chem..

[70] Chan P.F., Ang K.P., Hamid R.A. (2021). A Bismuth Diethyldithiocarbamate Compound Induced Apoptosis via Mitochondria-Dependent Pathway and Suppressed Invasion in MCF-7 Breast Cancer Cells. BioMetals.

[71] da Luz J.Z., Machado T.N., Bezerra A.G., de Oliveira Ribeiro C.A., Neto F.F. (2020). Cytotoxicity of Bismuth Nanoparticles in the Murine Macrophage Cell Line RAW 264.7. J. Mater. Sci. Mater. Med..

[72] Kobayashi J., Ikeda K., Sugiyama H. (2017). Cytotoxicity of Bismuth Compounds to Cultured Cancer Cells. J. Environ. Anal. Toxicol..

[73] Luo Y., Wang C., Qiao Y., Hossain M., Ma L., Su M. (2012). In Vitro Cytotoxicity of Surface Modified Bismuth Nanoparticles. J. Mater. Sci. Mater. Med..

[74] Song Q., Liu Y., Jiang Z., Tang M., Li N., Wei F., Cheng G. (2014). The Acute Cytotoxicity of Bismuth Ferrite Nanoparticles on PC12 Cells. J. Nanoparticle Res..

[75] Liu Y., Zhuang J., Zhang X., Yue C., Zhu N., Yang L., Wang Y., Chen T., Wang Y., Zhang L.W. (2017). Autophagy Associated Cytotoxicity and Cellular Uptake Mechanisms of Bismuth Nanoparticles in Human Kidney Cells. Toxicol. Lett..

[76] Abudayyak M., Öztaş E., Arici M., Özhan G. (2017). Investigation of the Toxicity of Bismuth Oxide Nanoparticles in Various Cell Lines. Chemosphere.

[77] Kim Y.S., Brechbiel M.W. (2012). An Overview of Targeted Alpha Therapy. Tumor Biol..

[78] Brechbiel M.W. (2007). Targeted Alpha-Therapy: Past, Present, Future?. Dalton Trans..

[79] Lima L.M.P., Beyler M., Oukhatar F., Le Saec P., Faivre-Chauvet A., Platas-Iglesias C., Delgado R., Tripier R. (2014). H_2_Me-Do2pa: An Attractive Chelator with Fast, Stable and Inert (Nat)Bi^3+^ and ^213^Bi^3+^ Complexation for Potential α-Radioimmunotherapy Applications. Chem. Commun..

[80] Chan S., Wang R., Man K., Nicholls J., Li H., Sun H., Chan G.C.F. (2019). A Novel Synthetic Compound, Bismuth Zinc Citrate, Could Potentially Reduce Cisplatin-Induced Toxicity Without Compromising the Anticancer Effect Through Enhanced Expression of Antioxidant Protein. Transl. Oncol..

[81] Jiang H., Hong Y., Fan G. (2022). Bismuth Reduces Cisplatin-Induced Nephrotoxicity Via Enhancing Glutathione Conjugation and Vesicular Transport. Front. Pharmacol..

[82] Brum J.M., Gibb R.D., Ramsey D.L., Balan G., Yacyshyn B.R. (2021). Systematic Review and Meta-Analyses Assessment of the Clinical Efficacy of Bismuth Subsalicylate for Prevention and Treatment of Infectious Diarrhea. Dig. Dis. Sci..

[83] Goldman R.D. (2013). Bismuth Salicylate for Diarrhea in Children. Can. Fam. Physician.

[84] Bowen A., Agboatwalla M., Pitz A., Salahuddin S., Brum J., Plikaytis B. (2019). Effect of Bismuth Subsalicylate vs Placebo on Use of Antibiotics among Adult Outpatients with Diarrhea in Pakistan: A Randomized Clinical Trial. JAMA Netw. Open.

[85] Peng J., Xiong Y., Lin Z., Sun L., Weng J. (2015). Few-Layer Bismuth Selenides Exfoliated by Hemin Inhibit Amyloid-β 1-42 Fibril Formation. Sci. Rep..

[86] Yang N., Sun H. (2011). Bismuth: Environmental Pollution and Health Effects. Encycl. Environ. Health.

[87] Bradley B., Singleton M., Po A.L.W. (1989). Bismuth Toxicity—A Reassessment. J. Clin. Pharm. Ther..

[88] Gao X., Wang Y., Peng S., Yue B., Fan C., Chen W., Li X. (2015). Comparative Toxicities of Bismuth Oxybromide and Titanium Dioxide Exposure on Human Skin Keratinocyte Cells. Chemosphere.

[89] Gao X., Zhang X., Wang Y., Fan C. (2016). Effects of Morphology and Surface Hydroxyl on the Toxicity of BiOCl in Human HaCaT Cells. Chemosphere.

[90] Dopp E., von Recklinghausen U., Hippler J., Diaz-Bone R.A., Richard J., Zimmermann U., Rettenmeier A.W., Hirner A.V. (2011). Toxicity of Volatile Methylated Species of Bismuth, Arsenic, Tin, and Mercury in Mammalian Cells in Vitro. J. Toxicol..

[91] Liman R. (2013). Genotoxic Effects of Bismuth (III) Oxide Nanoparticles by Allium and Comet Assay. Chemosphere.

[92] Larsen A., Martiny N., Stoltenberg M., Danscher G., Rungby J. (2003). Gastrointestinal and Systemic Uptake of Bismuth in Mice after Oral Exposure. Pharmacol. Toxicol..

[93] Sano Y., Satoh H., Chiba M., Okamoto M., Serizawa K., Nakashima H., Omae K. (2005). Oral Toxicity of Bismuth in Rat: Single and 28-Day Repeated Administration Studies. J. Occup. Health.

[94] Sano Y., Satoh H., Chiba M., Shinohara A., Okamoto M., Serizawa K., Nakashima H., Omae K. (2005). A 13-Week Toxicity Study of Bismuth in Rats by Intratracheal Intermittent Administration. J. Occup. Health.

[95] Dorso L., Bigot-Corbel E., Abadie J., Diab M., Gouard S., Bruchertseifer F., Morgenstern A., Maurel C., Chérel M., Davodeau F. (2016). Long-Term Toxicity of 213Bi-Labelled BSA in Mice. PLoS ONE.

[96] Omouri Z., Hawari J., Fournier M., Robidoux P.Y. (2018). Bioavailability and Chronic Toxicity of Bismuth Citrate to Earthworm Eisenia Andrei Exposed to Natural Sandy Soil. Ecotoxicol. Environ. Saf..

[97] He N., Li X., Feng D., Wu M., Chen R., Chen T., Chen D., Feng X. (2013). Exploring the Toxicity of a Bismuth-Asparagine Coordination Polymer on the Early Development of Zebrafish Embryos. Chem. Res. Toxicol..

[98] Fowler B.A., Sullivan D.W., Sexton M.J. (2015). Chapter 31—Bismuth. Vol. I, Handbook on the Toxicology of Metals.

[99] Pelepenko L.E., Janini A.C.P., Gomes B.P.F.A., de-Jesus-Soares A., Marciano M.A. (2022). Effects of Bismuth Exposure on the Human Kidney—A Systematic Review. Antibiotics.

[100] Hudson M., Mowat N. (1989). A Reversible Toxicity in Poisoning with Colloidal Bismuth Subcitrate. BMJ.

[101] Taylor E.G., Klenerman P. (1990). Acute Renal Failure after Colloidal Bismuth Subcitrate Overdose. Lancet.

[102] Playford R.J., Matthews C.H., Campbell M.J., Delves H.T., Hla K.K., Hodgson H.J., Calam J. (1990). Bismuth Induced Encephalopathy Caused by Tri Potassium Dicitrato Bismuthate in a Patient with Chronic Renal Failure. Gut.

[103] Huwez F., Pall A., Lyons D., Stewart M.J. (1992). Acute Renal Failure after Overdose of Colloidal Bismuth Subcitrate. Lancet.

[104] Akpolat I., Kahraman H., Arik N., Akpolat T., Kandemir B., Cengiz K. (1996). Acute Renal Failure Due to Overdose of Colloidal Bismuth. Nephrol. Dial. Transpl..

[105] Summers W.K. (1998). Bismuth Toxicity Masquerading as Alzheimer’s Dementia. J. Alzheimers Dis..

[106] Hruz P., Mayr M., Löw R., Drewe J., Huber G. (2002). Fanconi’s Syndrome, Acute Renal Failure, and Tonsil Ulcerations after Colloidal Bismuth Subcitrate Intoxication. Am. J. Kidney Dis..

[107] Reynolds P.T., Abalos K., Hopp J., Williams M.E. (2012). Bismuth Toxicity: A Rare Cause of Neurologic Dysfunction. Int. J. Clin. Med..

[108] Akinci E., Koylu R., Yortanli M., Gumus H., Koylu O., Altintepe L., Cander B. (2015). Acute Bismuth Intoxication: Acute Renal Failure, Tonsillar Ulceration and Posterior Reversible Encephalopathy Syndrome. Hong Kong J. Emerg. Med..

[109] Sampognaro P., Vo K.T., Richie M., Blanc P.D., Keenan K. (2017). Bismuth Subgallate Toxicity in the Age of Online Supplement Use. Neurologist.

[110] Disel N.R., Açikalin A., Sebe A., Gokel Y. (2017). Utilization of Plasmapheresis in the Management of Bismuth Intoxication with Acute Renal Failure. Saudi J. Kidney Dis. Transpl..

[111] Borbinha C., Serrazina F., Salavisa M., Viana-Baptista M. (2019). Bismuth Encephalopathy—A Rare Complication of Long-Standing Use of Bismuth Subsalicylate. BMC Neurol..

[112] Hogan D.B., Harbidge C., Duncan A. (2018). Bismuth Toxicity Presenting as Declining Mobility and Falls. Can. Geriatr. J..

[113] Yu C., Eustaquio N., Calello D.P., Ruck B.E., Nelson L.S., Santos C. (2019). Bismuth Subsalicylate Coagulopathy in a Patient with Chronic Liver Disease. J. Med. Toxicol..

[114] Sharma R.R., Cast I.P., Redfern R.M., O’Brien C. (1994). Extradural Application of Bismuth Iodoform Paraffin Paste Causing Relapsing Bismuth Encephalopathy: A Case Report with CT and MRI Studies. J. Neurol. Neurosurg. Psychiatry.

[115] Harris R.A., Poole A. (2002). Beware of Bismuth: Post Maxillectomy Delirium. ANZ J. Surg..

[116] Roest M.A.B., Shaw S., Orton D.I. (2002). Allergic Contact Otitis Externa Due to Iodoform in BIPP Cavity Dressings. Contact Dermat..

[117] Youngman L., Harris S. (2004). BIPP Madness; an Iatrogenic Cause of Acute Confusion. Age Ageing.

[118] Ovaska H., Wood D.M., House I., Dargan P.I., Jones A.L., Murray S. (2008). Severe Iatrogenic Bismuth Poisoning with Bismuth Iodoform Paraffin Paste Treated with DMPS Chelation. Clin. Toxicol..

[119] Atwal A., Cousin G.C.S. (2016). Bismuth Toxicity in Patients Treated with Bismuth Iodoform Paraffin Packs. Br. J. Oral. Maxillofac. Surg..

[120] Chen Y., Psaltis A.J., Curragh D.S., Selva D. (2018). Neurotoxicity Secondary to Bismuth Iodoform Paraffin Paste Packing in an Orbital Exenteration Cavity. Ophthalmic. Plast. Reconstr. Surg..

[121] Tan R., Neo S., Gan J., Fu E., Lim M.Y., Li H. (2021). Myoclonus from Intoxication by Bismuth Iodoform Paraffin Paste (BIPP) Nasopharyngeal Packing. Cureus.

[122] Matias M., Pinho J.O., Penetra M.J., Campos G., Reis C.P., Gaspar M.M. (2021). The Challenging Melanoma Landscape: From Early Drug Discovery to Clinical Approval. Cells.

[123] Guiard E., Lelievre B., Rouyer M., Zerbib F., Diquet B., Mégraud F., Tison F., Bignon E., Lassalle R., Droz-Perroteau C. (2019). Bismuth Concentrations in Patients Treated in Real-Life Practice with a Bismuth Subcitrate-Metronidazole-Tetracycline Preparation: The SAPHARY Study. Drug Saf..

