# Supplementary material for: Bioactive Bismuth Compounds: Is Their Toxicity a Barrier to Therapeutic Use?
Source: Int J Mol Sci. 2024 Jan 27;25(3):1600. doi: 10.3390/ijms25031600 (PMC10855265; doi:10.3390/ijms25031600)
Supplement: Supplementary file 1 [file ijms-25-01600-s001.zip › ijms-2814618-supplementary.pdf]

International Journal of *Molecular Sciences*

MDPI

Supplementary Material

Bioactive Bismuth Compounds: Is Their Toxicity a Barrier to Therapeutic Use?

Ângela Gonçalves [1], Mariana Matias [1], Jorge A. R. Salvador [2,3] and Samuel Silvestre [1,3,*]

[1] CICS-UBI—Health Sciences Research Centre, University of Beira Interior, Av. Infante D. Henrique, 6201-506 Covilhã, Portugal; angela.goncalves@ubi.pt (Â.G.); mariana.matias@fcsaude.ubi.pt (M.M.)
[2] Laboratory of Pharmaceutical Chemistry, Faculty of Pharmacy, University of Coimbra, 3000-548 Coimbra, Portugal; salvador@ff.uc.pt
[3] CNC—Centre for Neuroscience and Cell Biology, University of Coimbra, 3004-517 Coimbra, Portugal
* Correspondence: sms@ubi.pt

Table S1. Main pharmacological properties of bismuth compound according preclinical studies.

Bismuth compound	Main pharmacological properties	Reference
Bismuth(III) hydroxamate complexes	Antibacterial activity (*Helicobacter pylori*)	[44]
Bismuth-fluoroquinolone complexes	Antibacterial activity (*Helicobacter pylori*)	[46]
Bismuth(III) 5-sulfosalicylate complexes	Antibacterial activity (*Helicobacter pylori*)	[47]
Phenylbismuth(III) sulfonate complexes	Antibacterial activity (*Helicobacter pylori*)	[48]
Bismuth(III) flavonolate complexes	Antibacterial activity (*Staphylococcus aureus*, methicillin-*resistant Staphylococcus aureus*, and vancomycin-resistant *Enterococcus*, *Escherichia coli*, *Pseudomonas aeruginosa*)	[50]
Di-aryl bismuth phosphinates	Antibacterial activity (methicillin-resistant *Staphylococcus aureus*, vancomycin-resistant *Enterococcus*)	[51]
Bismuth thiolates	Antibacterial activity (methicillin-resistant *Staphylococcus aureus*)	[52]
Bismuth nanoparticles	Antibacterial activity (*Helicobacter pylori* or *Streptococcus salivarius* and *Enterococcus faecalis*)	[45,53]
Cyclic organobismuth, bearing a nitrogen or sulphur atom as an additional ring member	Antibacterial activity (*Staphylococcus aureus*, *Bacillus subtilis*, and *Enterococcus faecalis*)	[54]
Bismuth(III) phenyl pyrazolinates	Antibacterial (*Bacillus lichaniformis* and *Vibrio* spp) and antifungal (*Aspergillus niger* and *Penicillium*	[55]

	notatum) activities	
Bis(dialkyldithiocarbamato)diorganodithiophosphatobismuth(III) complexes	Antibacterial activity (*Staphylococcus aureus, Escherichia coli, Pseudomonas aeruginosa*)	[56]
Bismuth(III) salicylate and pyrazoline complexes	Antibacterial (*Staphylococcus aureus, Bacillus lichaniformis, Klebsiella pneumoniae, Vibrio* ssp, *Escherichia coli, Pseudomonas aeruginosa*) and antifungal (*Aspergillus niger* and *Penicillium notatum*) activities	[57]
Bismuth-norfloxacin complexes	Antibacterial activity (*Escherichia coli, Klebsiella pneumoniae, Staphylococcus aureus, Bacillus pumilis and Staphylococcus epidermidis*)	[58]
Triorganobismuth(V) biscarboxylates	Antileishmanial activity (*Leishmania tropica*)	[60]
Triarylbismuth dihalides and halobismuthanes	Antifungal activity (*Saccharomyces cerevisiae*)	[61]
Heterocyclicorganobismuth(III) based on a diphenyl sulfone scaffold	Antifungal activity (*Saccharomyces cerevisiae*)	[62]
Peptide–bismuth bicycles	Antiviral activity (Zika and West Nile)	[63]
Ranitidine bismuth citrate	Antiviral activity (SARS-CoV-2)	[35]
Bi-chlorodibenzo[*c,f*][1,5]thiabismocine	Anticancer activity	[66]
Tris[2-(*N,N*-dimethylaminomethyl)phenyl]-bismuthane	Anticancer activity	[67]
Bismuth 8-quinolinethiolates	Anticancer activity	[68]
Bismuth(III) dithiocarbamate complexes	Anticancer activity	[70]
Bismuth citrate	Anticancer activity	[30]
Bismuth subgallate	Anticancer activity	[72]
Bismuth nanoparticles	Anticancer activity	[65, 69, 73-75]
Bismuth subsalicylate	Anti-Alzheimer's disease activity	[85]
